# PICLS with human cells is the first high throughput screening method for identifying novel compounds that extend lifespan

**DOI:** 10.1186/s13062-024-00455-4

**Published:** 2024-01-23

**Authors:** Mohammad Alfatah, Yizhong Zhang, Arshia Naaz, Trishia Yi Ning Cheng, Frank Eisenhaber

**Affiliations:** 1 Bioinformatics Institute (BII), Agency for Science, Technology and Research (A*STAR), 30 Biopolis Street, Matrix #07-01, Singapore, 138671 Republic of Singapore; 2 Genome Institute of Singapore (GIS), Agency for Science, Technology and Research (A*STAR), 60 Biopolis Street, Genome #02-01, Singapore, 138672 Republic of Singapore; 3 LASA – Lausitz Advanced Scientific Applications gGmbH, Straße der Einheit 2-24, 02943 Weißwasser, Federal Republic of Germany; 4 School of Biological Sciences (SBS), Nanyang Technological University (NTU), Singapore, 637551 Republic of Singapore

**Keywords:** Chronological lifespan, High-throughput methods, Chemical screening, Human cells, 2,5-Anhydro-d-mannitol, Rapamycin, Anti-aging, Longevity

## Abstract

Gerontology research on anti-aging interventions with drugs could be an answer to age-related diseases, aiming at closing the gap between lifespan and healthspan. Here, we present two methods for assaying chronological lifespan in human cells: (1) a version of the classical outgrowth assay with quantitative assessment of surviving cells and (2) a version of the PICLS method (propidium iodide fluorescent-based measurement of cell death). Both methods are fast, simple to conduct, cost-effective, produce quantitative data for further analysis and can be used with diverse human cell lines. Whereas the first method is ideal for validation and testing the post-intervention reproductive potential of surviving cells, the second method has true high-throughput screening potential. The new technologies were validated with known anti-aging compounds (2,5-anhydro-d-mannitol and rapamycin). Using the high-throughput screening method, we screened a library of 162 chemical entities and identified three compounds that extend the longevity of human cells.

**Supplementary Information:**

The online version contains supplementary material available at 10.1186/s13062-024-00455-4.

## Introduction

Since 1950, life expectancy has drastically increased from 47 to 73 years. Despite the recent trend toward dwindling birth rates, the world population spiked from 2.9 billion in 1950 to 7.8 billion in 2020. This remarkable increase in human longevity results in a higher proportion of the population above 65 years of age—with almost one out of five in the most advanced countries [1–3]. However, the increase in the number of years lived is not a representation of the health status of the elderly. There is a remarkable gap of almost 10 years between the healthspan, which is the period essentially free from diseases, and the actual lifespan [1–3]. Elders tend to have costly chronic diseases such as cancer, neurodegenerative and cardiovascular diseases and other metabolic syndromes, which severely hamper their quality of life and ability to work [4–10]. As such, this puts tremendous pressure on the healthcare system and the working population. Aging has become a global challenge in the twenty-first century. Gerontology research has to address those challenges. The application of yet-to-discover anti-aging drugs and food additives, which aim at reducing chronic disease progression and increasing healthspan, is one of the possible measures.

For identifying interventions that delay aging and extend cellular lifespan, the budding yeast *Saccharomyces cerevisiae* has been widely used as a eukaryotic model system [11,12]. There are two important assays to measure the cellular lifespan in yeast; replicative lifespan (RLS) and chronological lifespan (CLS) [13–18]. RLS refers to the number of divisions undergone by a cell before entering into the post-mitotic phase, for example, in the case of human stem cells. CLS refers to the length of time that a cell can survive in a non-dividing state, modelling for the aging of post-mitotic cells such as nerve and muscle cells.

The PICLS high-throughput screening (HTS) method, an advanced method for accessing CLS-related interventions in yeast, reduces costs and time spent compared with classical outgrowth methods by about a factor of a hundred [19]. Although the majority of yeast genes and pathways being regulated in aging-related processes share significant similarities to those in humans, there are still critical differences in the aging of yeast and humans [16]. Hence, it would be both beneficial and essential to conduct similar experiments directly in human cell cultures.

The current gold standard in the field [20], is a yeast-like chronological lifespan (CLS) assay with human cells, as established by Leontieva and Blagosklonny [21]. This method represents the first reported instance of horizontally transferring the CLS assay from yeast to human cell lines. The validation of this approach was carried out using inhibitors of mTOR, PI-3K, and MEK, specifically rapamycin, LY294002, and U0126, respectively, in HCT116 and HT-p21-9 cell lines. In accordance with their protocol, human cells enter the post-mitotic stage by nutrient deprivation under selected anti-aging experimental conditions. Survival of post-mitotic cells is then measured by outgrowth in a fresh medium. I.e., exhausted medium (together with floating dead cells) is aspirated. Attached cells are trypsinized and transferred to 6-well plates with fresh medium. They are incubated for seven days before determining the cell viability via counting the number of grown colonies using crystal violet staining. This method was applied to prove the anti-aging effect of rapamycin [21] as well as the flavonoid 4,4′-dimethoxychalcone to promote CLS in cells of several species including humans [22].

However, this method requires a long time for the experiment. At the outgrowth stage, the 6-well plates need seven days to reach sufficient cell counts to be detected by the crystal violet assay. If primary fibroblasts were used instead of immortal cell lines, it would require an even longer growing period. Further, the method is not suitable for cells with a low adherent ability to the culture plate. The rinsing of cells before trypsinization and crystal violet staining would result in varying levels of cell loss due to detachment, hence, leading to uncontrollable variations in the results. This method is also not suitable for high-throughput screening as the number of compounds and concentrations tested are restricted by the plate capacity. The crystal violet assay generates image data for qualitative visual comparison. However, the experimental data from the assay is typically presented as an image of stained cell patches. Due to variations in sizes and irregular shapes of different human cell types, it becomes challenging to quantify the number of stained cells and account for cell loss during repeated washings in the assay. Consequently, the qualitative nature of the data fails to capture minor changes in cell viability induced by attempted anti-aging interventions. Therefore, it is crucial to develop a method that (i) minimizes the need for cell washing, (ii) utilizes quantitative tools correlating to the number of cells, (iii) suitable for HTS, (iv) applies to various human cell types, and (v) generates quantitative data for analysis.

In this paper, we report two refined methods of assessing cell viability in human cells that are inspired by our PICLS HTS technology for assessing CLS-related interventions for yeast [19]. We continue to use the 96-well plate together with the microplate reader for quantitative data collection. The first method uses the PrestoBlue (PB) assay and enables detailed analysis of cell viability testing based on an outgrowth assay. In contrast, the second method with propidium iodide (PI) staining enables quick and easy spectroscopic measurement of the number of dead cells in wells.

Compared to crystal violet method, prestoblue assay minimizes the requirement of cell washing, the chemical used provides a fluorescent signal that correlates to the number of cells. On the other hand, the PI method does not require any washing and the chemical also produces a fluorescent signal that correlates to the number of dead cells. The fluorescent signal detected from the methods above is a more accurate representation of the results compare to irregular cell patches. Thus, the latter variant (PICLS with Human cells) is genuinely suitable for large-scale HTS aimed at identifying compounds that prolong CLS.

## Results and discussion

### Evaluation of cellular lifespan using the outgrowth crystal violet assay

2,5-Anhydro-d-mannitol (2,5-AM), a fructose analogue, has been known for its effect in inhibiting gluconeogenesis and cancer [23,24]. Recently, this compound has been reported to increase the CLS of *S. cerevisiae* [19]. However, its anti-aging activity in human cells if any was unknown. We wished to evaluate the anti-aging effect of 2,5-AM in human cells. After a literature search, we found only one method applicable for CLS measurement in human cell lines using a yeast-like CLS methodology [21].

In this approach, an outgrowth assay is used to determine the number of live cells in the 6-well experiment plate using crystal violet staining. Crystal violet is a triarylmethane dye that binds to ribose molecules such as DNA [25]. The crystal violet-based outgrowth method relies on the detachment of adherent cells from the culture plate during cell death. Hence, dead cells would be washed away with the D10 medium and only live cells would remain attached to the culture plate. These live cells were then stained with crystal violet dye and accessed qualitatively.

**Fig. 1:**
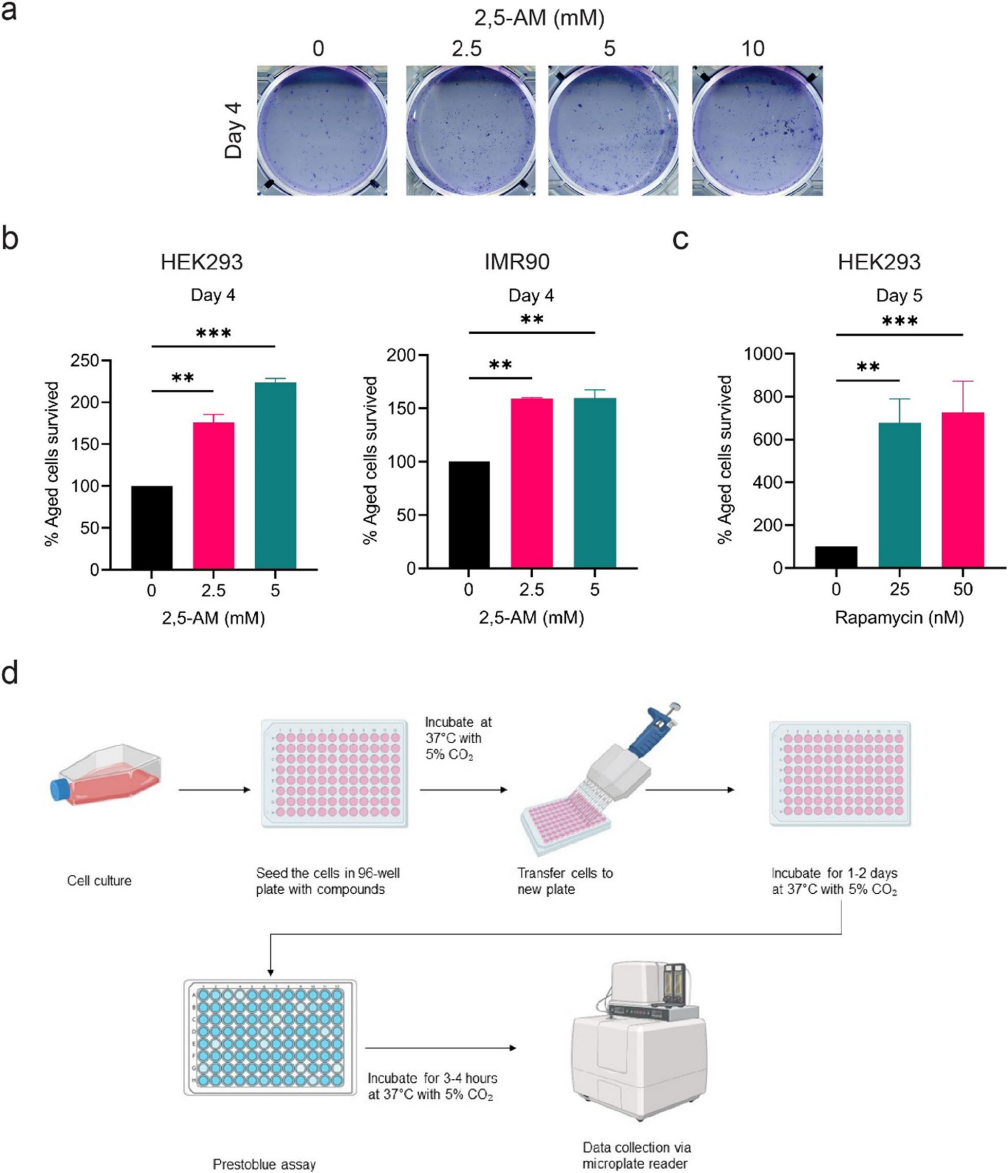
Outgrowth assays determined the anti-aging activity of 2,5-AM and rapamycin in human cells. **a** CLS determination by qualitatively assessing the cells’ ability to proliferate. Cells were plated without (control) or with 2,5-AM compound treatment at several concentrations. On the fourth day after cells were seeded and treated, cells were trypsinized and 2% (4 µl out of 200 µl) of the cells were transferred to a 6-well experiment plate with fresh D10 medium. The experiment plate was incubated at 37 °C incubator with 5% CO_2_ for seven days before staining with the Crystal Violet Assay. **b** CLS determination by quantitatively assessing the cells’ ability to proliferate with 2,5-AM treatment. The same cells were used as in (**a**), 10% (20 µl out of 200 µl) of the trypsinized cells were transferred to a 96-well experiment. **c** CLS determination by the outgrowth assay with rapamycin treatment. A similar experiment procedure was done as in (**b**) with 2,5-AM. **d** Schematic diagram illustrating the experimental procedure of the outgrowth assay. **b**–**c** were analyzed by ordinary one-way ANOVA followed by Dunnett’s multiple comparisons tests. Results are plotted as mean ± SD. *P ≤ 0.05; **P ≤ 0.01; ***P < 0.001

We examined the cell viability of 2,5-AM treated cells at different concentrations using the crystal violet-based yeast-like CLS assay. We observed that after seven days of outgrowth, 2,5-AM is able to increase cellular lifespan in the HEK293 cell line (Fig. 1a). Similar results can be observed on the Day 8 culture but not on the Day 6 culture (Additional file 1: Fig. S1a). This may be caused by the variation in washing steps during the crystal violet staining. Nonetheless, these results prove that 2,5-AM can increase cellular lifespan in the human cell line, confirming its conserved anti-aging properties over vast eukaryote taxonomic ranges.

#### Benefits

The outgrowth crystal violet assay offers a cost-effective and adaptable method for quantitatively assessing cellular lifespan in human cell lines. Its simplicity, ability to visually confirm live cells, and relevance to studying conserved anti-aging properties make it a valuable method.

#### Limitations

However, this assay requires a long time for cellular outgrowth. There is a risk of detaching cells during multiple washing steps in the staining. It is not suitable for HTS analysis of multiple compounds/concentrations/treatments. The qualitative nature of the data could obscure analysis being ambiguous or misleading at a low number of surviving cells.

### Evaluation of cellular lifespan using outgrowth PrestoBlue assay

In order to overcome some of the limitations mentioned above, we have developed a new method that requires less time, has a lesser experimental error and, most importantly, generates quantitative data for CLS analysis of human cells. We have utilised an anti-aging compound, 2,5-AM, to validate the efficiency of our new approach based on the outgrowth PrestoBlue assay.

PrestoBlue (PB) is a resazurin-based reagent used to detect cell viability and traces of cytotoxicity in the medium. It produces results that can be detected both colourimetrically and fluorometrically. It is permeable and nontoxic to the cells, hence, enabling the measurement of cell viability over a period of time without cell lysis. The resazurin component is reduced to form resorufin by live cells, resulting in a visible change from blue colour to red colour in the reagent, at the same time developing fluorescence, which can be easily detected by a microplate reader [26]. The PB reagent is highly sensitive; it detects even a small number of cells (a couple of hundred). It allows convenient fast cell detection and data collection. The PB assay is easy to perform as the reagent is commercially available as a ready-to-use solution [26].

In the outgrowth assay using human cell lines, 2,5-AM treatment significantly increases the cellular lifespan, which results in more live cells that were able to proliferate in the experiment plate. For the HEK293 cell line, the 2.5 mM treatment group had 76.31% more viable cells compared to the untreated group (Fig. 1b). To note, the 5 mM treatment group had even 123.7% more surviving cells relative to the untreated group (Fig. 1b).

We also tested the anti-aging effect of 2,5-AM in the human lung primary fibroblast cells, IMR90. The primary fibroblast cells are widely used in aging research as they have a finite lifespan, and they better mimic the organism’s aging process compared to immortal cell lines [27,28]. An anti-aging effect similar to that for HEK293 was also observed for the IMR90 cells. The 2.5 mM treatment group had 59.05% more surviving cells and the 5 mM treatment group had 59.89% more viable cells compared to the untreated group (Fig. 1b).

We also evaluate the cellular outgrowth for 5% (10 µl) transferred to the experiment plates for the HEK293 and IMR90 cell experiments. We found a pattern of anti-aging activities similar to the results when 10% of cells were transferred (Additional file 1: Fig. S1b). Thus, these results suggest that the time for outgrowth can be shortened by increasing the number of transferred cells without affecting the results.

We further tested the PB-based outgrowth method with rapamycin, an established anti-aging compound in multiple models, including the HT-p21-9 cell assay where it increases cellular lifespan [21]. We find that rapamycin treatment significantly increases the lifespan of HEK293 cells (Fig. 1c). This further confirms the robustness of the method to identify and evaluate potential anti-aging compounds.

Our new outgrowth assay (Fig. 1d) allows us to determine cellular lifespan in a short time with a significant throughput capacity. The detection of viable cells using the PB reagent provides quantitative data, which can be computationally further evaluated. Notably, other resazurin-based reagents such as the MTT, XTT and alamarBlue reagent can replace PB as well as any efficient colourant that stains surviving cells.

#### Benefits

The outgrowth PB assay offers a streamlined and efficient method for evaluating cellular lifespan, addressing limitations of traditional assays. With reduced experimental time and potential errors, it generates quantitative data, enhancing precision in CLS analysis for human cells. PB's versatility and sensitivity enable convenient, user-friendly assessments, proving applicable across different cell lines. The assay’s ability to confirm anti-aging effects, demonstrated with 2,5-AM and rapamycin, underscores its reliability in identifying potential anti-aging compounds. Notably, the method allows for computational analysis, further enriching data interpretation and providing a robust tool for anti-aging research with improved efficiency and accuracy.

#### Limitations

The outgrowth assay requires aspiration of medium, washing and trypsinization like the outgrowth crystal violet assay. These steps introduce multiple sources of errors to the experiment such as cell detachment during aspiration and washing, potential problems during trypsinization or the transfer of live cells. To note, the outgrowth assay depends on the cells’ ability to proliferate in the fresh medium after days of compound treatment. Hence, if the treatment results in a decline in cell proliferation, cells will not proliferate even if they are alive, giving a false negative result. For example, calorie restriction has been shown to increase cellular lifespan but reduce proliferation [29]. Thus, the outgrowth assay is not suitable for the detection of cellular lifespan in this scenario. Moreover, inefficiency in detecting surviving cells with low metabolic activity is also the main criticism towards the PB assays and other related assays that measure cytotoxicity via NADH-dependent cellular oxidoreductase enzymes [26,30].

### Evaluation of cellular lifespan using the propidium iodide assay as in the PICLS method

In order to overcome the limitations of the outgrowth approaches, we adapted the PICLS method (PI-based CLS measurement in yeast) for usage with human cells [19]. This method does not require the outgrowth procedure, including aspiration, washing, trypsinization and transferring cells to a new plate. We have utilised 2,5-AM and rapamycin to validate the efficiency of our new method for quantification of cellular lifespan, using the PI fluorescence-based method.

PI is a nucleic acid intercalating dye that is used to detect necrotic or apoptotic cells. It is impermeable to live cells with intact membranes, but able to penetrate dead or cells with damaged membranes to bind to DNA and RNA [31]. Upon intercalating between the bases, the quantum signal of PI increases drastically, which can then be detected by the microplate reader.

**Fig. 2:**
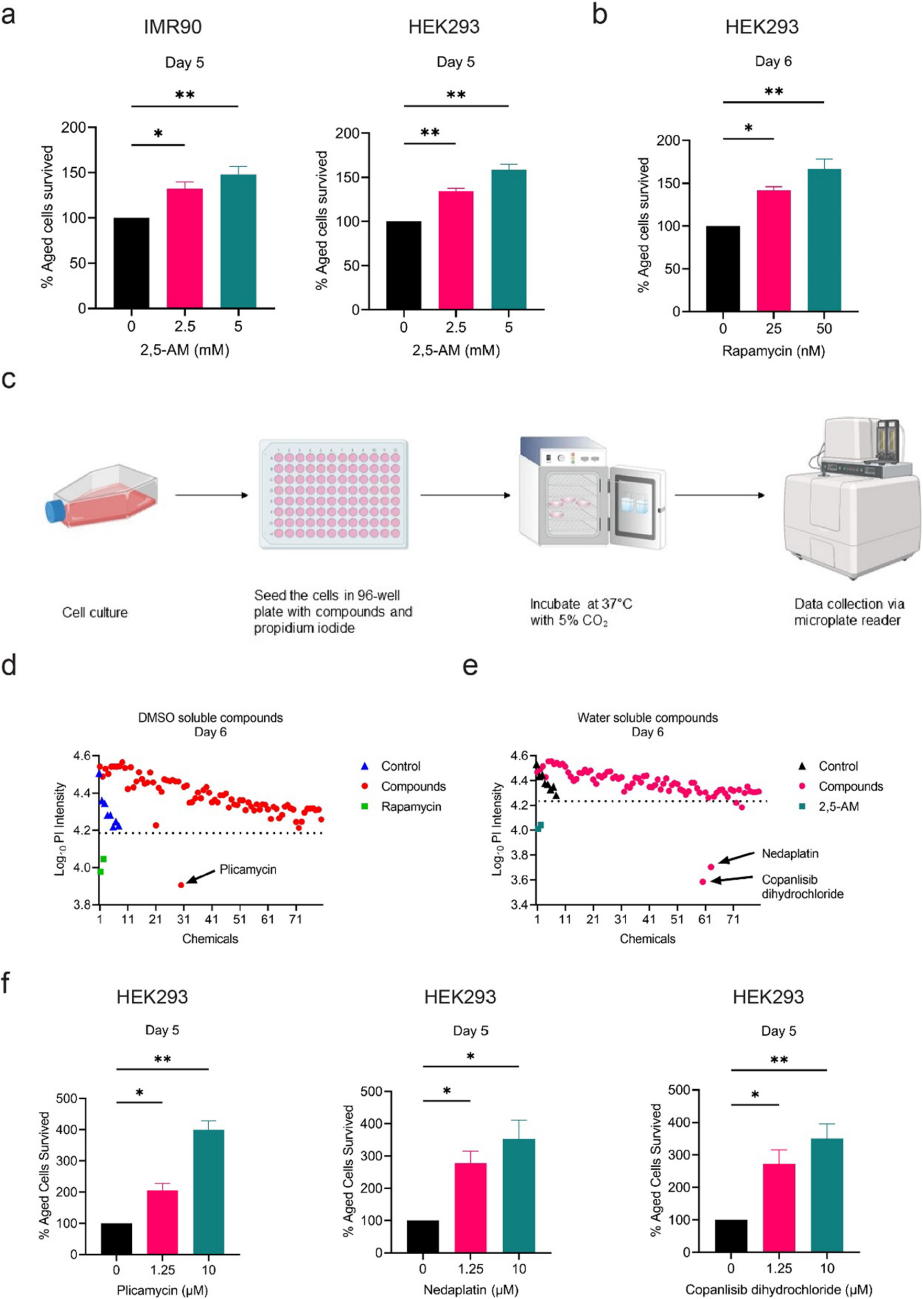
CLS determination using the PI assay (PICLS with human cells). **a** CLS determination by PI assay with 2,5-AM treatment. Cells were seeded in a 96-well plate with PI (5 µg/ml) dissolved in the D10 medium. Fluorescence reading was taken on the fifth day after seeding. **b** CLS determination by the PI assay with rapamycin treatment, a similar experimental procedure was performed as in (**a**). **c** Schematic diagram illustrating the experimental procedure of the cell viability assay. DMSO (**d**) and water-soluble (**e**) compounds screening using PICLS assay determines the potential anti-aging compounds that extend cellular lifespan. 50 nM rapamycin (**d**) and 20 mM 2,5-AM (**e**) were used as positive controls. Graphs depict data from Day 6 results. **f** CLS determination by cell viability assay with compounds found positive in (**d**–**e**). **a**–**b**, **f** were analyzed by ordinary one-way ANOVA followed by Dunnett’s multiple comparisons tests. Results are plotted as mean ± SD. *P ≤ 0.05; **P ≤ 0.01

In the PI assay using IMR90 cells, 2,5-AM treatment significantly increases the cellular lifespan, resulting in a lower percentage of cell death. Both the 2.5 mM and the 5 mM treatment groups show 32.33% and 48.08% more live cells compare to the untreated control, respectively (Fig. 2a). We also found that 2,5-AM increases cellular lifespan in the HEK293 cells, compared to the untreated group. The 2.5 mM and 5 mM treatment experiment show 34.39% and 58.7% more live cells, respectively (Fig. 2a). Likewise, rapamycin application shows a similar trend in the HEK293 cell line (Fig. 2b). We also evaluated the cellular viability using 2 µg/ml of PI in HEK293 and IMR90 cells. We found a similar pattern of anti-aging activities despite the methodical variation (Additional file 1: Fig. S1c).

#### Benefits

The new PI assay (Fig. 2c) is more economical and reduces variations introduced in the outgrowth methods as it requires one plate per experiment only. The assay is easy to perform as no additional steps are required after the experiment plate has been cultured, and readings can be taken at multiple age time points with the same samples on a microplate reader. The PI assay measures the amount of cell death. Hence, it is independent of the cell’s ability to proliferate. Therefore, it is also suitable for compounds or procedural interventions that suspend cell proliferation.

These advantages make the PICLS-type assays highly compatible with high-throughput compound screening requirements. It is not just restricted to the CLS measurement alone. It can be used for senolytic compound screening with senescent-induced cells or as anti-cancer compound screening with cancer cell lines under nutrient-limiting conditions. Thus, there is more potential in this cell viability assay with the usage of different cell lines. The PICLS-type HTS method is potentially valuable in many additional ways for drug discovery and biopharmaceutical research.

#### Limitations

The specificity of the PI assay for measuring cell death limits its applicability to scenarios focused on understanding the impact of compounds on cellular lifespan through cell death mechanisms. This method may not be suitable when simultaneous assessment of both cell death and proliferation dynamics is necessary for a comprehensive analysis of cellular lifespan. Despite efforts to reduce experimental variations, results obtained with the PI assay may still be subject to variability introduced during the experimental process, necessitating careful execution and standardization to ensure reliable outcomes.

### High-throughput chemical screening using PICLS assay with human cells

We utilised the PI-based method to identify novel anti-aging compounds that prolong CLS. We screened a total of 162 compounds (including some FDA-approved drugs and natural products). We find that the log10 value of the PI fluorescent reading for the various control samples ranges from 4.22 to 4.53. Hence, compounds with clearly lower log10 PI fluorescent readings should be viewed as extending CLS. In addition to the known anti-aging compounds rapamycin and 2,5-AM that were added to the screen as positive controls, further three compounds exhibited a low log10 PI fluorescent reading.

Among the DMSO-soluble compounds, plicamycin was detected to increase CLS (reading of 3.91 on Day 6; see Fig. 2d). From the set of water-soluble compounds tested, copanlisib dihydrochloride and nedaplatin are demonstrated to be anti-aging (log10 PI fluorescent readings of 3.59 and 3.70, respectively; Fig. 2e).

These compounds were further validated with different concentrations using PI assay. For all three compounds, plicamycin, nedaplatin and copanlisib dihydrochloride, treatment significantly increases the lifespan of HEK293 cells. The 1.25 µM and the 10 µM plicamycin application samples show 105.7% and 299.8% more live cells compare to the untreated control, respectively (Fig. 2f). Similarly, the 1.25 µM and the 10 µM nedaplatin treated cultures show 177.2% and 253.7% more live cells while the samples with 1.25 µM and the 10 µM copanlisib dihydrochloride show 172.3% and 250.6% more live cells (Fig. 2f).

Plicamycin, copanlisib dihydrochloride and nedaplatin are potential anti-cancer agents [32–34]. However, the potential of these compounds in extending CLS has not yet been reported. To note, the known anti-cancer mechanism of action of these drugs reviewed below might not be centrally relevant for the cellular lifespan extension in the experimental setting tested here.

Plicamycin is identified to bind to the GC-rich sequences in DNA, it prevents transcription factors from complexing with promoters and inhibits RNA synthesis [35]. Nedaplatin (a derivative of cisplatin) binds to DNA and forms a cross-link. This inhibits DNA synthesis and replication [33].

Copanlisib dihydrochloride is the inhibitor of phosphoinositide 3-kinase (PI3K; PI3K activation enables the tumour to evade immune detection) [32]. Notably, PI3K positively regulates the TORC1 (Target of Rapamycin Complex 1) pathway [36]. We think that this actually might cause the CLS extension. TORC1 is a conserved eukaryotic protein complex from yeast to humans that couples the presence of nutrients with DNA replication, transcription and translation [37–39]. TORC1 promotes cellular anabolic processes such as the synthesis of nucleotides, proteins and it inhibits the catabolic process, including oxidative phosphorylation and autophagy [37,40–43].

TORC1 positively regulates the aging process [4,5,37]. The clinically approved drug rapamycin inhibits TORC1 and it extends the murine lifespan and healthspan [44–46]. Rapamycin (sirolimus) and its analogues everolimus (Afinitor) and temsirolimus (torisel) are currently applied for a few chronic diseases, including cancer counting. Rapamycin is in clinical trials for its use as an anti-aging therapeutic [47]. Mechanistically, we hypothesize that plicamycin, copanlisib dihydrochloride and nedaplatin might exert their lifespan extending properties through modulation of TORC1 signalling network or via directly influencing translation of its component-encoding genes. We are in the progress to explore the detailed mechanisms of the longevity activity of these compounds.

## Conclusion

The human cell-based outgrowth assay remains ideal for evaluating the cellular lifespan effect of selected anti-aging interventions based on the live cell’s ability to proliferate in a suitable medium. Despite the limitations in cell detachment and unsuitable for compounds that suppress cell proliferation, it is nonetheless a very informative method, especially as the PB version with quantitative signal reading described in this work. Further, we find that our new version of the PICLS assay, measuring the degree of cell death under compound treatment, is highly suitable for HTS compound screening in human cells. It is simple to set up and cost-effective, it is also not limited by the type of compounds used. The PI assay can be conducted with a large variety of human cell lines, such as cells from various human tissues but also cancer and senescence-induced cell lines.

While the viability assays utilized in our study (PB and PI dyes) are well-known in the field for testing cell viability, our innovative approach involves their application to determine the chronological lifespan of human cells. This novel utilization of PB and PI has not been previously reported in the context of assessing cell longevity. The PI-based method offers a rapid and efficient readout (within ~ 15 min) using a microplate reader, a cost-effective device widely available in most laboratories. This economical approach is particularly well-suited for large-scale screening of chemical agents to identify potential anti-aging compounds. Various substances, such as natural products, metabolites, FDA-approved drugs, controls, or dilutions, can be conveniently accommodated on a single plate, and multiple plates can be processed simultaneously. A notable improvement over existing methods is that the developed PI method allows for screening a greater number of chemicals using a 96-well plate compared to the traditional 6-well plate, enhancing test efficiency by 16-fold and reducing time consumption by 85%, providing a viability readout within one day, as opposed to the 7 days required with the crystal violet method [21].

The PICLS HTS recovers both known anti-aging drugs as well as detects new candidate compounds (pliamycin, copansilib and nedaplatin). Further, PICLS can be useful to access the anti-cancer or senolytic potential of large sets of compounds or interventions. We recommend using these two cell viability measuring methods in conjunction, with the PICLS technology for large-scale compound screening and the PB outgrowth assay to validate the finding and evaluate the cellular replicative potential in more detail. We think that these methods will be largely applicable in cellular lifespan research as well as in other areas of biopharmaceutical, drug discovery and toxicity research.

## Methods

### Human cell lines, growth media and cell culture

HEK293 human cell line (ATCC, Manassas, Virginia, United States of America) and IMR90 fibroblast cell line (Coriell Institute, Camden, New Jersey, United States of America) were cultured in the standard D10 medium, consisting of high-glucose DMEM (HyClone #SH30022.01) supplemented with 10% FBS (Gibco™ #10270106) and 1% Penicillin Streptomycin Solution (Gibco™ #15140122). All cells were cultured in a humidified incubator with 5% CO_2_ at 37 °C.

### Chemical treatment to cell culture

The stock solution of rapamycin was prepared in dimethyl sulfoxide (DMSO), followed by serial dilution to form various concentrations used in the experiment. The final DMSO concentration was kept at 0.05% in all experiments. The stock solution of 2,5-anhydro-D-mannitol was prepared in a D10 medium. The chemical library used in screening was tested at 1 µM concentrations. Controls used were the same as the solvent applied, DMSO or water.

### The Leontieva and Blagosklonny 2011 CLS assay in human cells

This assay was performed as previously described [21]. In brief, 80,000 cells were seeded in a 96-well culture plate with or without compound treatment. On Day 4, Day 6 and Day 8 after seeding, floating dead cells were aspirated with the exhausted D10 medium. Then, attached cells were gently washed with 1X Phosphate-buffered saline (PBS) to remove D10 medium residue and treated with 0.2 ml of 0.25% trypsin. Cells were gently mixed by pipetting and a 2% aliquot (4 µl) was transferred to 6-well experiment plates with fresh D10 medium. After seven days of growth in a 37 °C incubator with 5% CO_2_, the crystal violet assay was performed on the test plate to determine the viability of cultured cells [25]. Crystal violet experiment plates were imaged using a scanner.

### CLS determination in human cell lines using the PB outgrowth assay

The seeding, washing and trypsinization process is similar to that in the previous paragraph. 80,000 cells were seeded in a 96-well culture plate with or without compound treatment. At different age time points after seeding, floating dead cells were aspirated with exhausted D10 medium, cells were gently washed with 1X PBS to remove D10 medium residue and treated with 0.2 ml of 0.25% trypsin. Cells were gently mixed by pipetting and a 5% aliquot (10 µl) or 10% aliquot (20 µl) were transferred to 96-well experiment plates with fresh D10 medium. After one day of growth in a 37 °C incubator with 5% CO_2_, the cell number was assessed with PrestoBlue™ Cell Viability Reagent (Invitrogen™ #A13261) according to the manufacturer's protocol. In brief, we performed a tenfold dilution upon the cell viability reagent with growth D10 medium. We aspirated the D10 medium from the 96-well plate and replaced it with the diluted PrestoBlue reagent. After incubation in a 37^O^C incubator with 5% CO_2_ for 3–4 h, the reading was taken with a microplate reader at 560 nm excitation and 590 nm emission (BioTeck Synergy MX).

### CLS determination in human cell lines using the PI assay

The assay was performed by preparing the cells with PI (2 µg/ml or 5 µg/ml) in a D10 medium. 80,000 cells were seeded in a 96-well culture plate with or without compound treatment. The culture plate was used as the experiment plate and was protected from light throughout the experiment, growing in a 37 °C incubator with 5% CO_2_. At age time points, reading was taken with a microplate reader at 535 nm excitation and 617 nm emission (BioTeck Synergy MX).

### Data analysis

Statistical analysis and graphs were completed with GraphPad Prism v.9.4.1 software. Results were analysed with one-way ANOVA and two-way ANOVA tests, followed by multiples comparison by Dunnett’s post hoc test or Sidak’s post hoc test. In the graph plots, *P* values were shown as *P < 0.05, **P < 0.01, ***P < 0.001, and ****P < 0.0001. These were considered significant (n.s. indicates non-significant comparison).

### Supplementary Information

**Additional file 1:** Figure S1.

## Data Availability

The data that supports the findings of this study are available in the supplementary material of this article.
