# Supplementary material for: PICLS with human cells is the first high throughput screening method for identifying novel compounds that extend lifespan
Source: Biol Direct. 2024 Jan 23;19:8. doi: 10.1186/s13062-024-00455-4 (PMC10804585; doi:10.1186/s13062-024-00455-4)
Supplement: Supplementary file 1 — Additional file 1: Figure S1. [file 13062_2024_455_MOESM1_ESM.docx]

**Supplementary material**

**PICLS with human cells is the first high throughput screening method that identifies novel compounds that extending lifespan**

Mohammad Alfatah^1^*, Yizhong Zhang^1^, Arshia Naaz^2^, Trishia Yi Ning Cheng^1^ and Frank Eisenhaber^1,3,4^

1. Bioinformatics Institute (BII), Agency for Science, Technology and Research (A*STAR), 30 Biopolis Street, Matrix #07-01, Singapore 138671, Republic of Singapore
2. Genome Institute of Singapore (GIS), Agency for Science, Technology and Research (A*STAR), 60 Biopolis Street, Genome #02-01, Singapore 138672, Republic of Singapore
3. LASA – Lausitz Advanced Scientific Applications gGmbH, Straße der Einheit 2-24, D-02943 Weißwasser, Federal Republic of Germany
4. School of Biological Sciences (SBS), Nanyang Technological University (NTU), Singapore 637551, Republic of Singapore

*To whom the correspondence should be addressed.

Email: alfatahm@bii.a-star.edu.sg (Mohammad Alfatah)


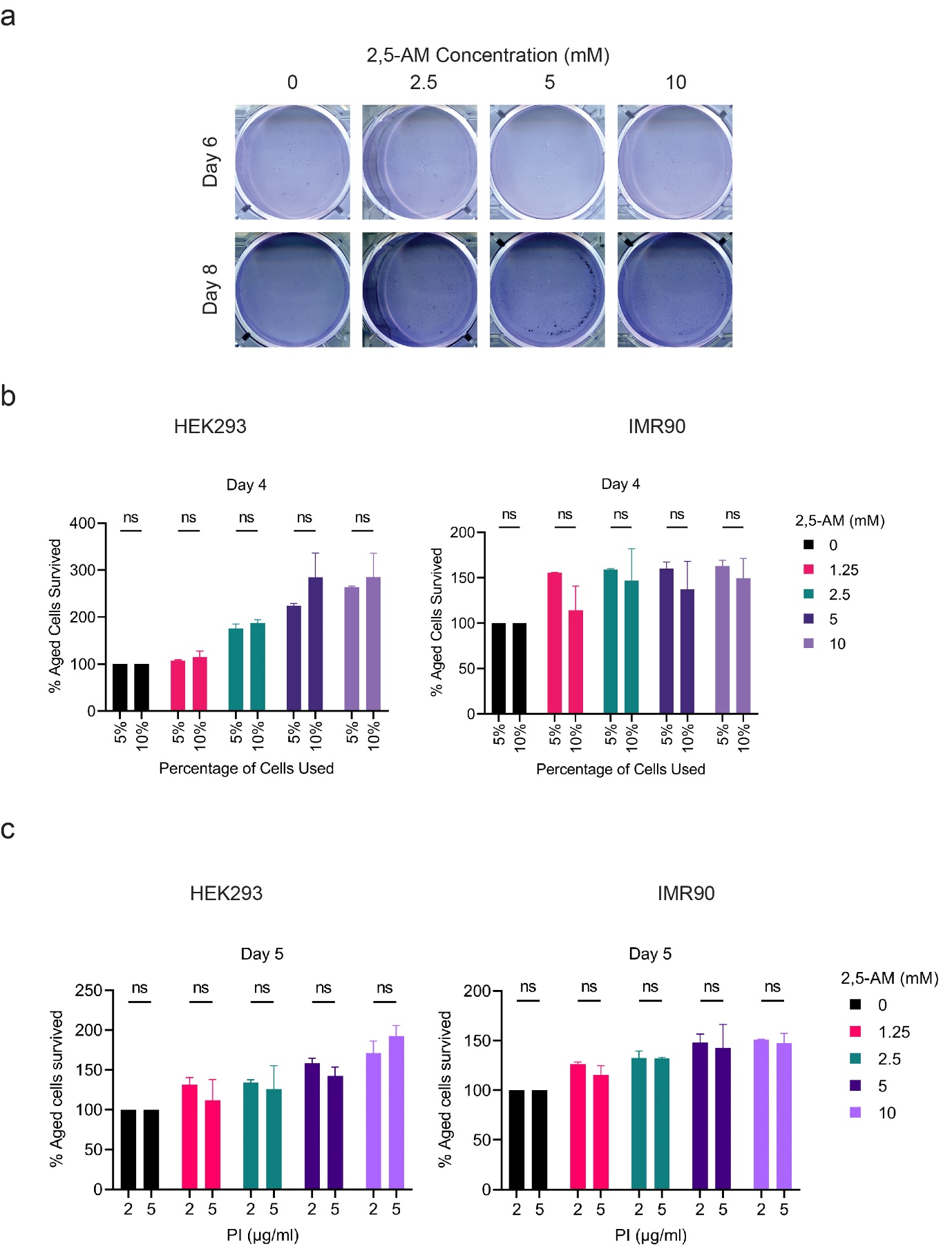


**Figure S1. CLS determined by outgrowth and PI assays**

(a) CLS determination by qualitatively assessing the cell’s ability to proliferate. Cells were plated without (control) or with 2,5-AM compound treatment at several concentrations. On Day 6 and Day 8, after cells were seeded and treated, cells were trypsinized and 2% (4 µl out of 200 µl) of the cells were transferred to a 6-well experiment plate with fresh D10 medium (Supplement for Figure 1a). The experiment plate was incubated at 37^O^C with 5% CO_2_ for seven days before staining with the Crystal Violet Assay. (b) CLS determination by quantitatively assessing the cell’s ability to proliferate with 2,5-AM treatment. Comparing the cellular outgrowth for 5% (10 µl) and 10% of cells transferred to the experiment plate in HEK293 and IMR90 cells (Supplement for Figure 1b). (c) Comparing the cellular viability using 2 µg/ml PI or 5 µg/ml of PI in HEK293 and IMR90 cells (Supplement for Figure 2a). (b-c) Data were analysed with two-way ANOVA followed by Sidak’s post hoc test. Results are plotted as mean ± SD, n.s was non-significant.
